# Resting quantitative myocardial perfusion—essential clinical coronary pathophysiology for preventing non-beneficial revascularization in cardiomyopathy

**DOI:** 10.1093/ehjci/jeaf158

**Published:** 2025-05-24

**Authors:** K Lance Gould, Nils P Johnson

**Affiliations:** Weatherhead PET Center for Preventing and Reversing Atherosclerosis, Division of Cardiology, Department of Medicine, McGovern Medical School, University of Texas, 6431 Fannin Street, Houston, TX 77030, USA; Memorial Hermann Hospital, 6411 Fannin Street, Houston, TX 77030, USA; Weatherhead PET Center for Preventing and Reversing Atherosclerosis, Division of Cardiology, Department of Medicine, McGovern Medical School, University of Texas, 6431 Fannin Street, Houston, TX 77030, USA; Memorial Hermann Hospital, 6411 Fannin Street, Houston, TX 77030, USA

**This editorial refers to ‘The prognostic interplay between PET-derived resting myocardial blood flow and left ventricular ejection fraction’, by A. Sayed *et al*., https://doi.org/10.1093/ehjci/jeaf132.**

In this issue of European Heart Journal Cardiovascular Imaging, Sayed *et al*.^1^ report rest and stress myocardial blood flow (MBF) in mL/min/g, myocardial flow reserve (MFR) as the stress/rest ratio, and ejection fraction (EF) by electrocardiogram (ECG)-gated PET images in 8089 patients with outcomes of all-cause death (466) and hospitalizations for heart failure (819). Both high resting myocardial blood flow (MBFrest) and low MFR were associated with an increased risk of death or congestive heart failure (CHF) and significant interaction with EF. At a given constant MFR across all EFs, high MBFrest carried a greater risk of death or CHF than predicted by EF alone.

The authors are to be complimented on interesting quantitative PET perfusion in a large cohort. However, this Editorial integrates comprehensive coronary pathophysiology explaining their observations with significant clinical implications. Resting MBF is autoregulated downward from the high coronary flow capacity needed for hard exertion. Autoregulation^2^ includes multiple variables including acute pressure-rate product, sympathetic tone, myocardial contractility, age, gender, obesity, endothelial function, left ventricular hypertrophy (LVH), LV wall stress, diffuse coronary artery disease (CAD), diastolic stiffness, microvascular dysfunction, the transmural perfusion gradient^3^ and blood volume. Moreover, resting MBF does not change significantly, before vs. after revascularization.^4^

In view of multifactorial autoregulation, this study population fits the well-established continuum of low MFR and low EF incurring the greatest risk of death/CHF up to high MFR and high EF incurring the lowest risk as seen in Editorial Figure 1 adapted from Sayed *et al*. Figure 4. However, Editorial Figure 1 also shows well-known exceptions with increasing death/CHF for increasing resting perfusion up to 1.45 mL/min/g for an EF of 50% (A) with parallel decreased MFR (B) reciprocally related to resting perfusion.

PET examples in *Figure 1* illustrate the wide spectrum of MBF or perfusion metrics due to diffuse non-obstructive CAD, LVH, microvascular dysfunction, non-ischaemic cardiomyopathy, or predominant cardiomyopathy mixed with bystander CAD without clinical ischaemia or angina or during PET stress imaging or relation to EF. These different pathophysiologies lumped together for a given EF, show a paradoxical high risk of death/CHF for high resting perfusion in subsets of cases. However, their different mechanism requires integrated comprehensive physiologic analysis of all perfusion metrics for clinical understanding and management.

**Figure 1:**
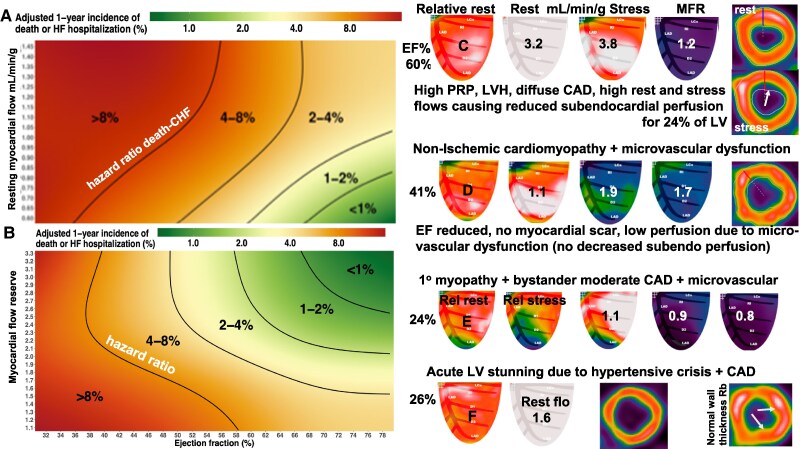
Heat maps of (*A*) resting perfusion, (*B*) MFR and EF (reproduced from Sayed *et al*^1^). PET examples, Case C through Case F, see text for details.

*Figure 1* Case C is an asymptomatic woman with dyslipidemia, hypertension, resting BP 159/59, heart rate 98, and LVH. The high-pressure-rate product and LVH incur very high-stress MBF through mild calcific multiple coronary stenosis by angiogram causing globally reduced subendocardial perfusion for 24% of the LV with 1.5 mm ST depression during dipyridamole stress without angina.^3^ Despite high-stress MBF, MFR as the ratio of MBFstress/MBFrest is reduced by high resting MBF. The high risk of death/CHF is due to risk factors, high pressure-rate product (PRP), diffuse CAD and LVH causing high rest MBF that is a bystander marker without independent or primary diagnostic value or risk. High rest perfusion, however, indicates that revascularization will not improve MBFrest. Bypass grafts to such high-flow arteries typically close. More intense medical treatment of risk factors is indicated.

Case D is a woman with resting and exertional dyspnoea, dyslipidemia, hypertension, diabetes, renal failure, atrial fibrillation, and calcific diffuse CAD by angiogram. Resting MBF is increased, stress MBF and MFR are reduced but well above ischaemic thresholds without reduced subendocardial perfusion thereby indicating predominant microvascular dysfunction limiting stress MBF and MFR.^3^ The EF is reduced in the absence of myocardial scar indicating non-ischaemic cardiomyopathy with microvascular dysfunction and bystander diffuse calcific non-obstructive CAD, all associated with high risk of death/CHF and bystander high resting MBF lacking independent diagnostic value or risk. Revascularization will not improve high resting MBF or low-stress MBF due to microvascular disease or improve EF due to primary non-ischaemic cardiomyopathy.

Case E is an asymptomatic man for pre-surgical clearance for colon cancer, chronic blood pressure 87/55, and abnormal SPECT scan. Resting PET relative images show a small apical non-transmural scar with moderately larger border zones during dipyridamole stress. Rest MBF is mildly elevated, stress MBF is severely reduced, and MFR is 0.8 in distal left anterior descending (LAD) distribution indicating myocardial steal and collateralization without angina or ST changes. The EF is severely reduced out of proportion to the small scar indicating predominant non-ischaemic cardiomyopathy with bystander moderate flow limiting CAD but without ischaemia.

Depending on clinical judgment and informed patient preferences, the PET findings for Case E suggest that revascularization has a low probability of benefit since (i) a low EF of 24% is reduced out of proportion to the size of the scar indicating predominant non-ischaemic cardiomyopathy with high resting perfusion in addition to bystander CAD. (ii) Revascularization will not further benefit or increase the current high resting MBF of 1.1 mL/min/g or prevent plaque rupture. (iii) Bypass graft to arteries with high resting MBF commonly close. (iv) Patient has no clinical angina or angina or STΔ during stress imaging. (iv) Revascularization has a low probability of improving EF, CHF, or 10-year survival for predominant non-ischaemic cardiomyopathy. (v) Proceeding with surgery for colon cancer without coronary intervention incurs an increased but lowest overall risk of this patient.

Case F is a woman inpatient for hypertensive crisis with a blood pressure of 210/80, heart rate of 114, cardiac index of 1.5 L/min, resting ECG with 2 mm ST depression, decompensated heart failure, atrial fibrillation, and EF of 20–25% while on intravenous (IV) nicardipine and IV nitroglycerine for blood pressure control during urgent resting PET for viability. Prior arteriogram reported moderate left main and three-vessel CAD. PET relative rest images showed no scar (all viable) with EF of 26% by ECG-gated PET perfusion images, very high resting MBF of 1.6 mL/min/g (normal 0.7), and thin-walled, dilated LV suggesting global severely reduced subendocardial perfusion. For comparison, the insert for another subject shows normal LV wall thickness for Rb-82 with two papillary muscles (white arrows) without subendocardial ischaemia.

For Case F, the low EF is due to acute hypertensive myocardial stunning with high rest flow due to high-pressure-rate product demand and IV coronary vasodilators (nicardipine + nitroglycrine) increasing coronary blood flow through narrowed arteries that lower intracoronary coronary pressure. The combination of high systemic systolic pressure, low coronary perfusion pressure, and tachycardia with short diastolic filling time caused severe global subendocardial ischaemia with a remaining thin subepicardial rim. Without knowledge of the blood pressure, heart rate, IV vasodilators, and ST changes, the PET images for Case F are comparable to dilated non-ischaemic cardiomyopathy rather than acute hypertensive myocardial stunning. With medical management, to control blood pressure and heart rate, tapering vasodilators, cardiac output increased to 3.0 L/min with the patient stable enough for percutaneous intervention (PCI) of left main stenosis. EF increased to 49% within days. *The critical lesson*: Comprehensive, coronary pathophysiology integrating clinical circumstances and quantitative PET perfusion are essential for PET-physiologic guidance for optimal management.

Cases E and F show the critical value of high resting perfusion with low EF without myocardial scar indicating most commonly the predominant non-ischaemic cardiomyopathy without sufficient flow limiting CAD or ischaemia indicating medical management without interventions. While rare, acute hypertensive stunning as for Case F may have PET images like non-ischaemic cardiomyopathy but correct interpretation requires incorporation of the clinical pathophysiology into the PET interpretation.

From our large database of 12 238 PET scans, *Figure 2A* shows EF as inversely related to the size of the myocardial scar as % of LV^4^ for differentiating non-ischaemic from ischaemic-scar cardiomyopathy. Of all PET scans, the red area of the plot identifies the 3% of PET scans with a reduced EF <50% caused by the scar. The green area of the plot classifies 8% of all PET scans with a reduced EF <50% out of proportion to the amount of scar. The white area between the green and red represents 3% of all PET scans with EF <50% that is only partially explained by the scar. Among PET scans with an EF <50% at rest, ‘infarct cardiomyopathy’ (red area) accounts for 21% of these cases. In another 20% (white area), scar partially accounts for the EF <50%. In the remaining 59% (green area) of PET scans with EF <50%, baseline LV function is reduced beyond what can be accounted for by scar size and severity—in other words, a predominately non-infarct cardiomyopathy (green area). Moreover, EF is poorly related to resting or stress myocardial perfusion in this large dataset.^4^

**Figure 2:**
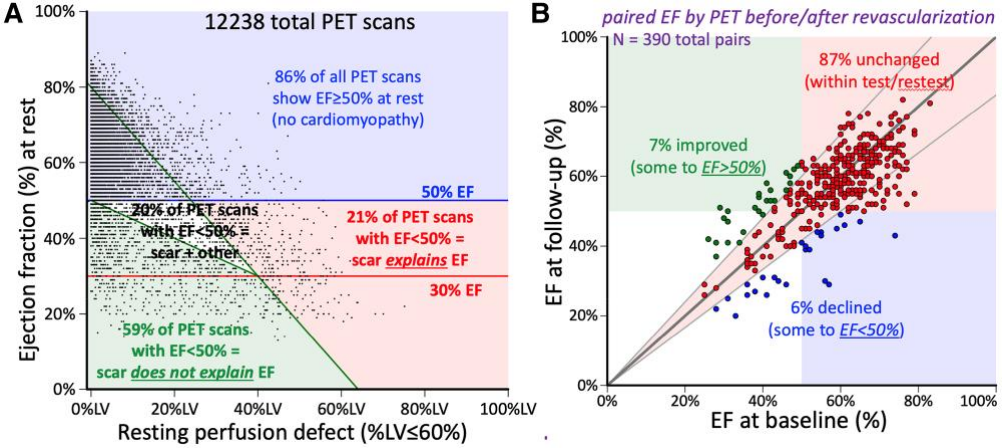
Relation of scar size to EF (adapted from Johnson and Gould^4^).

*Figure 2B* plots EF before and after revascularization in 390 paired PET images with ECG-gated EF of perfusion images in the same patient.^4^ The great majority, 87% had no change in EF that remained within ±10% with only 7% improved and 6% worsened EF from before to after revascularization. The few cases with improved LV function after revascularization typically have myocardial scar of less than 15% of LV, severely reduced coronary flow capacity for greater than 25% of LV, and resting EF ≥ 35%. Our data suggesting that most reduced EF is due to predominantly non-ischaemic cardiomyopathy with bystander CAD are consistent with randomized trials showing no improvement in survival or of EF. In our database, transmural viable stunned or hibernating myocardium is rare, less than 1%, while a subepicardial viable rim with fixed subendocardial scar greater than 50% of the transmural LV wall is common that typically does not improve LV function after revascularization (*Figure 2* reproduced with permission from Johnson and Gould^4^).

Quantitative stress perfusion (MBFstress), coronary flow reserve (MFR), and their combination as coronary flow capacity predict a high risk of death or major adverse coronary events (MACE) that is reduced by revascularization.^3^ In addition, scar size and resting MBF are essential for avoiding non-beneficial revascularization incurring significant risk in patients with low EF most commonly due to due to predominant non-ischaemic cardiomyopathy and bystander CAD.

## Data Availability

Data for this Editorial will be made available in de-identified format on request to one of the authors with an explanation for its use or analysis.
